# The declining trend in HIV prevalence from population-based surveys in Cameroon between 2004 and 2018: myth or reality in the universal test and treat era?

**DOI:** 10.1186/s12889-023-15374-8

**Published:** 2023-03-13

**Authors:** Cavin Epie Bekolo, C Kouanfack, J Ateudjieu, ET Bechem, SA Ndeso, N Tendengfor, DS Nsagha, SP Choukem

**Affiliations:** 1 Department of Public Health, Faculty of Medicine and Pharmaceutical Sciences, University of Dschang, PO Box 96, Dschang, Cameroon; 2 Department of Botany and Plant Physiology, Faculty of Sciences, University of Buea, Buea, Cameroon; 3 Department of Public Health and Hygiene, Faculty of Health Sciences, University of of Buea, Buea, Cameroon; 4 Department of Internal Medicine and Specialities, Faculty of Medicine and Pharmaceutical Sciences, University of Dschang, Dschang, Cameroon

**Keywords:** HIV Prevalence, Universal antiretroviral treatment, Sexual behaviour, Cameroon

## Abstract

**Background:**

HIV remains an epidemic of major public health importance in Cameroon but a decline in HIV prevalence has been observed according to population-based surveys conducted in 2004, 2011 and 2018. We sought to review current evidence for declining HIV prevalence despite increasing survival owing to ‘universal test and treat’ and to explore the reason for the decrease, particularly the role of behavioural change.

**Methods:**

We conducted a secondary analysis using HIV prevalence, behavioural and social determinants data of the Demographic and Health Survey Program databases. Trend lines were fitted to data that were available for a minimum of three points in time during the 1991–2018 period. Regression coefficients associated p-values and 95% confidence intervals were obtained using Microsoft Excel software.

**Results:**

Overall adult HIV prevalence decreased significantly from 5.4% (95%CI: 4.8-6.0) in 2004 to 4.3% (95%CI: 3.8–4.8) in 2011 and further down to 2.7% (95%CI: 2.3–3.1) in 2018 at a rate of about 1.4% every septennium (β = -1.4, R² = 0.98, p = 0.03). Yet, the number of persons surviving with HIV increased from about 0.05 million in 1991 to 0.5 million in 2018 corresponding to an increase in access to antiretroviral therapy from less than 10% to universal coverage of 80% respectively. Concurrent reductions in risky sexual behaviours were observed: a delayed sexual debut by one year, decreased sexual violence by 7%, decreased polygamous unions by 16%, decreased multiple sexual partners by 15.3% and increased condom use by 26.3%.

**Conclusion:**

The observed decline in HIV prevalence is statistically valid and reflects the observed decline in risky sexual behaviour that need to be sustained by the National HIV programme. Though universal access to ART has increased the number of persons surviving with HIV, this has not led to an increased prevalence of HIV in a setting with a rising population.

## Introduction

The human immunodeficiency virus (HIV) infection and the consequently acquired immunodeficiency syndrome (AIDS) constitute the leading cause of morbidity and death in Cameroon, accounting for 11.5 per cent of the disease burden and 14.2 per cent of deaths in 2013 [1, 2]. Overall adult HIV prevalence continues to decrease, moving from 5.4% to 2004 to 4.3% in 2011, 3.4% in 2017 and 2.7% in 2018 [3–6]. Cameroon thus has the highest HIV infection rates in West Central Africa (WCA). These data are important to both donor agencies funding HIV control programmes in Cameroon, and the government trying to bring about changes because they need to know whether their efforts are having an impact. It is important to understand changes in the incidence and prevalence of HIV to plan for the scale of future problems and to evaluate the effectiveness of current national strategies to limit the spread of infection. To make confident statements about the course of the HIV epidemic, we require confidence in the quality of data on the infection levels and in our ability to interpret changes in prevalence and incidence [7]. The decline in the prevalence of HIV observed amongst young men and women in Cameroon suggests some success in stemming the spread of HIV. However, when reductions in the prevalence of HIV are observed at a population level, it is intuitive to raise questions about whether the HIV prevalence declines are genuine or are the result of measurement bias. If real, there are additional questions regarding whether the prevalence declines are the result of a decline in incidence or due to rising mortality rates overtaking the rate of new infections. Furthermore, it is important to understand whether the observed changes are a mere reflection of the natural progression of the epidemic, a product of changes in behaviour, or a product of interventions such as ‘the universal test and treat all that has led to a scaling up of antiretroviral therapy (ART). ART has in turn transformed HIV infection from a rapid killer to a manageable chronic disease with a different epidemiological pattern [8, 9]. HIV prevalence data have been used to monitor trends in the HIV epidemic, but the rapid improvements in providing ART to people in need and the resulting increase in survival times are making it more difficult to rely on prevalence data only [10, 11]. To attempt to provide suitable answers to these questions, we reviewed the evidence from the last three Cameroon Demographic and Health Surveys (CDHS) that collected data on HIV prevalence. We aimed to explore the validity and interpretation of observed trends in HIV prevalence and incidence; develop a better understanding of observed epidemiological patterns; and guide the evaluation of changes in HIV prevalence.

## Data and methods

Data were drawn from the Demographic and Health Survey (DHS) website (www.dhsprogram.com) which reports nationally representative household surveys after obtaining permission from the DHS Program. We conducted a secondary analysis of the Cameroon DHS data collected from nationally representative household-based National HIV Prevalence, Incidence, Behaviour and Communication surveys completed in Cameroon in 1991, 1998, 2004, 2011 and 2018. The DHS Program uses a multi-stage cross-sectional design to select random sample clusters from a national sampling frame, usually from the national population census. Within the selected clusters, a full listing of all households is made before the survey and a systematic random sample of households is taken. During the main fieldwork, eligible women, and men, usually aged 15–49 and 15–59 years, respectively, are selected for HIV testing. An individual is only considered absent after three call-back visits. HIV testing is done using dried blood samples (DBS) samples of capillary blood from a finger prick, collected on special filter paper. DBS specimens are less painful, less invasive to collect and easier to store and transport than venous blood samples. A well-recognised central laboratory is identified to process the DBS samples for HIV testing after a careful assessment.

We selected twelve indicators of sexual behaviour recommended for monitoring and reporting changes in behaviour over time [12]. These indicators are median age at first sexual intercourse among women aged 25–49, percentage of young people aged 15–24 who have had sex before age 18, physical or sexual violence committed by husband/partner, the proportion of literate women, the proportion of women who are in polygamous unions, the median age at first marriage among women aged 25–49, men/women receiving an HIV test and receiving test results in the last 12 months, percentage of pregnant women aged 15–49 who received counselling, testing and results on HIV during prenatal care, percentage of young men/women aged 15–24 years who reported having had sexual intercourse with more than one partner in the past 12 months, percentage of young men/women aged 15–24 years who had more than one partner in the past 12 months and reported having used a condom during the last sex act, percentage of young men aged 15–24 years who had more than one partner in the past 12 months and reported having used a condom during the last sex act.

Complementary data were obtained from the UNAIDS website (https://www.unaids.org/en/resources/documents/2022/HIV_estimates_with_uncertainty_bounds_1990-present). We extracted National HIV estimates between 1990 and 2022 on prevalence, incidence, deaths, and ART coverage from the epidemiological spreadsheet made freely available for download. These estimates are derived from the Spectrum Projection Package (SPP) which uses the Estimation and Projection Package (EPP) model or the projection workbook to provide trends in HIV prevalence. It also requires assumptions about the epidemiology of HIV, including the ratio of female: to male prevalence, the distribution of infection by age, the distribution of the time from infection until AIDS death, and the effect of HIV on fertility [13].

Simple linear regression analyses were conducted to describe the trend in HIV prevalence as the response variable and behavioural indicators as the independent variables over time. Trend lines were fitted to data that were available for a minimum of three points in time during the 1990–2022 period. Regression coefficients (β), coefficients of determination (R²), associated p-values and 95% confidence intervals were obtained using Microsoft Excel software.

## Results

### Observed changes in HIV prevalence according to DHS data from 2004 to 2018

**Fig. 1:**
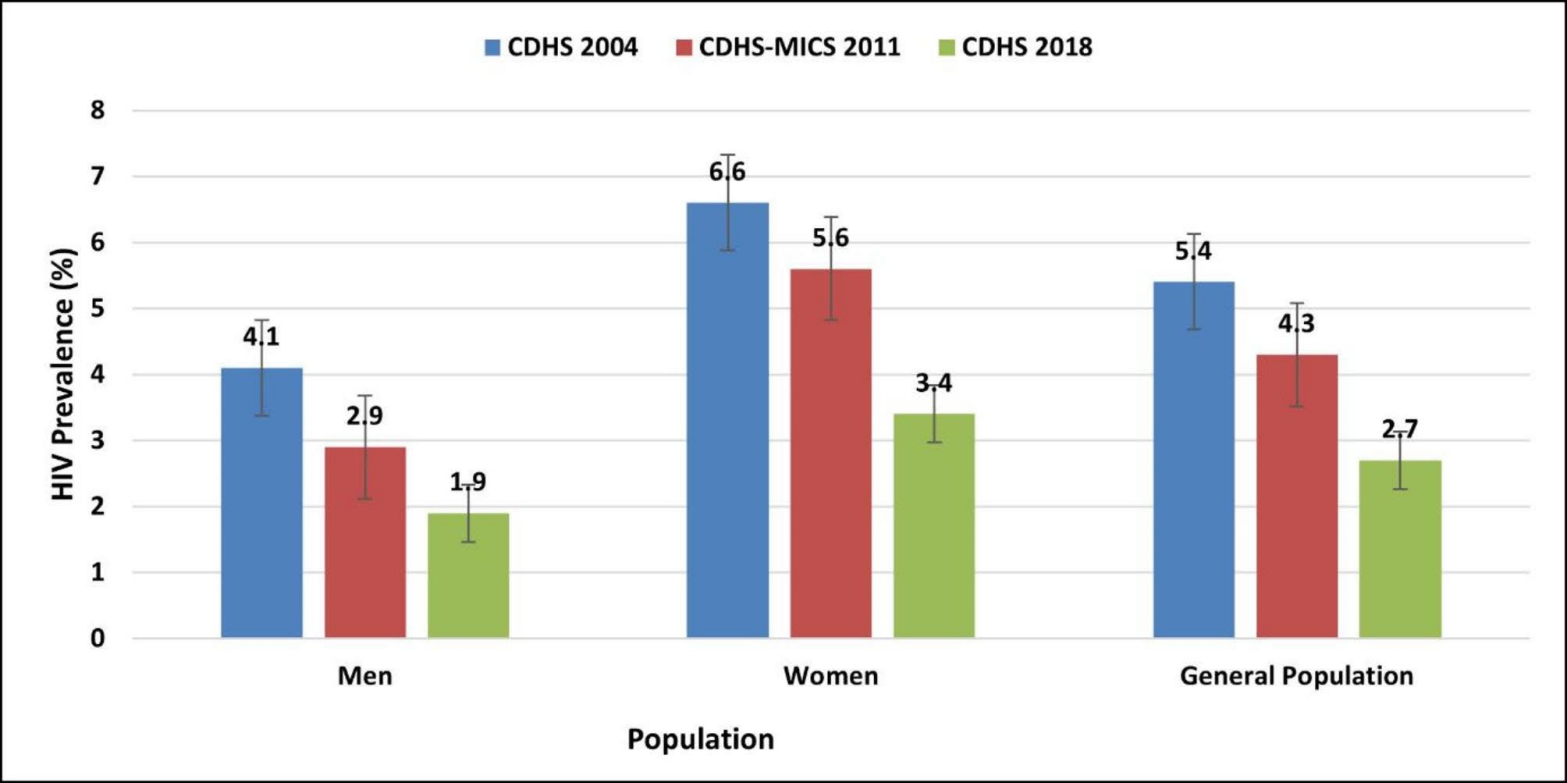
The trend in the prevalence of HIV in Cameroon from the last three population-based surveys between 2004 and 2018

In the past two decades, Cameroon has conducted three national population-based HIV surveys based on The Demographic and Health Surveys (DHS) Program to collect, analyse, and disseminate accurate and representative data on the state of the HIV epidemic in 2004, 2011 and 2018. Overall adult HIV prevalence decreased steadily, moving from 5.4% (95%CI: 4.8-6.0) in 2004 to 4.3% (95%CI: 3.8–4.8) in 2011 and further down to 2.7% (95%CI: 2.3–3.1) in 2018 at a rate of about 1.35% every septennium (β = -1.4, R² = 0.98, p = 0.03) (Fig. 1). Prevalence among women was approximately twice that of men (3.4% vs. 1.9% in 2018). HIV prevalence was higher among key populations (KP) at 24.3% (95%CI: 15,1–32,9) for female sex workers (FSWs) and 20.7% (95%CI: 3,9–43,3) for men who have sex with men (MSM) in 2016. HIV prevalence was higher in urban areas (2.9%) compared to rural areas (2.4%). HIV prevalence among adults 15–49 years varied by region in Cameroon, from a low of about 1% in the Far North to 5.6% in the East and 5.8% in the South Regions.

### Observed changes in HIV prevalence according to spectrum data from 1990 to 2022

**Fig. 2:**
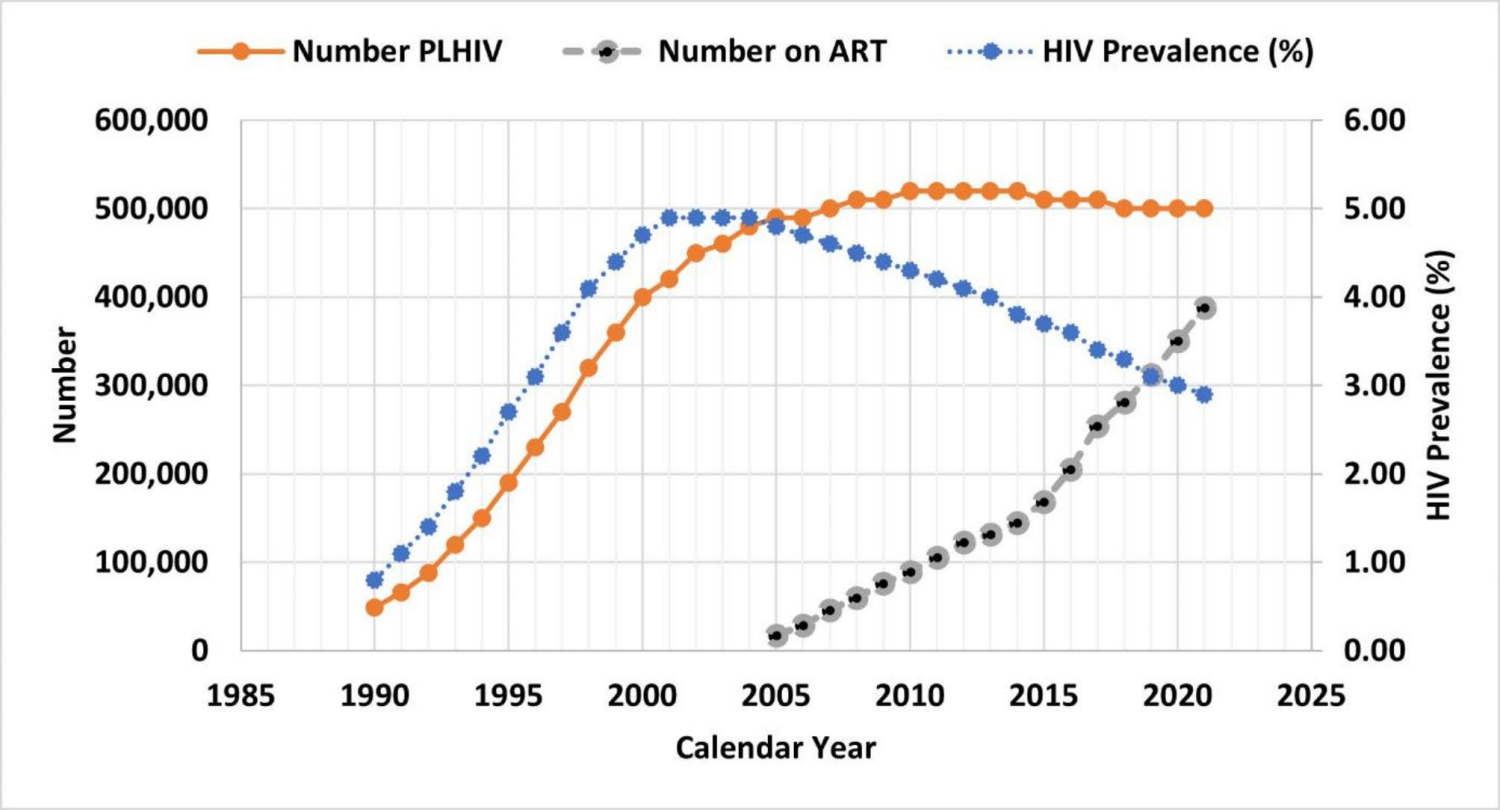
Adult HIV modelled prevalence, PLHIV and PLHIV receiving antiretroviral therapy in Cameroon between 1990 and 2022

**Fig. 3:**
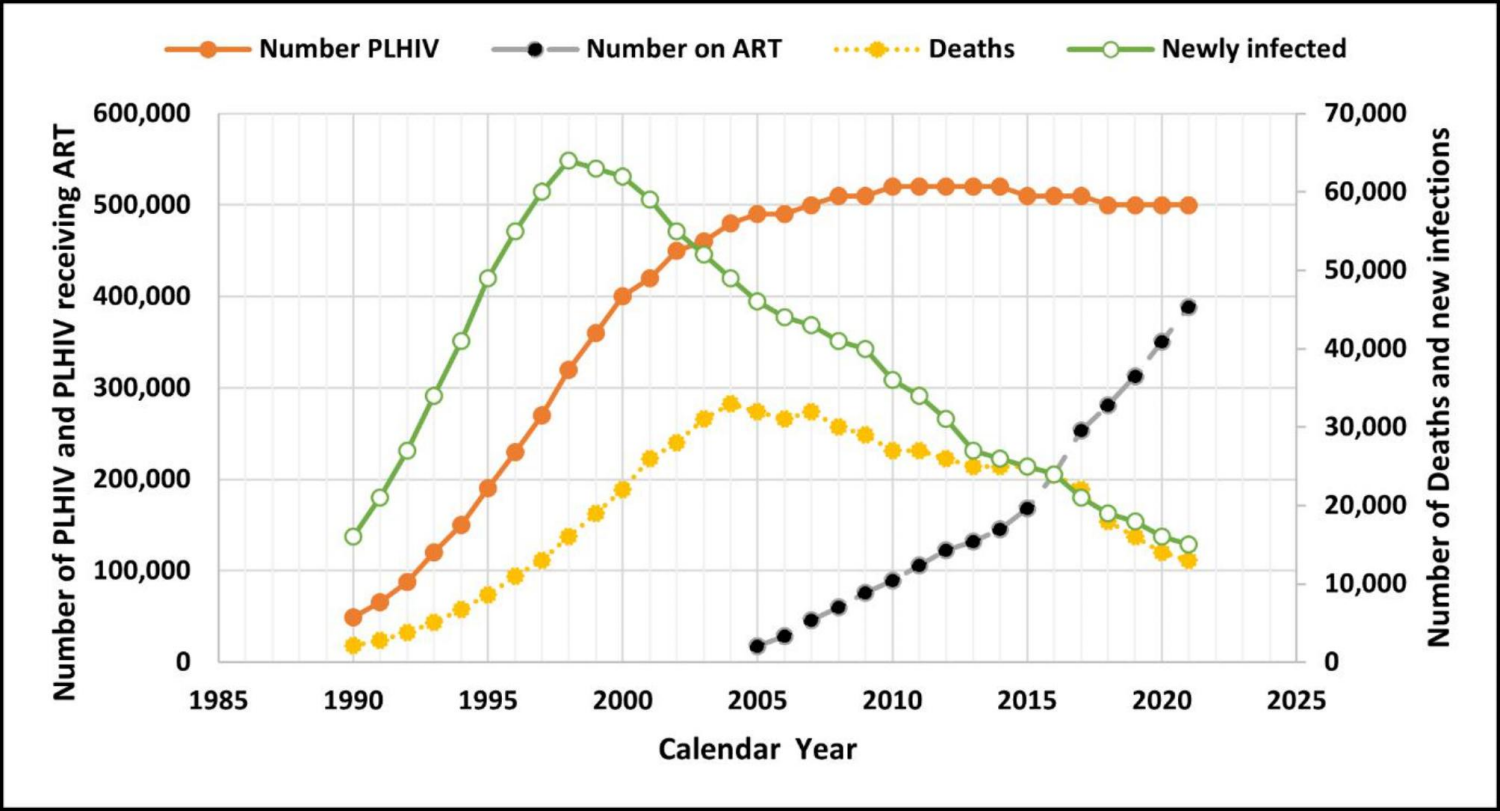
Incidence to mortality ratio in Cameroon between 1990 and 2020

Data from EPP show that the number of persons living with HIV (PLHIV) and the subsequent HIV prevalence increased rapidly from the 1990s reaching a peak in the early 2000s but while HIV prevalence started declining sharply from 2005, the number of PLHIV remained virtually stable thereafter (Fig. 2). Similarly, estimates of HIV incidence peaked around 1998 and then declined gradually thereafter. Mortality also peaked after the peak in incidence around 2004 and then started to decline thereafter but at a slower rate than the incidence rate (incidence/mortality ratio of 0.84). The exponential phase corresponded with the era of no ART while the decline phase matched the era of ART (Fig. 3).

### Observed changes in sexual behaviour from 1991 to 2018

As illustrated in Table 1, the trend in risky sexual behaviours between 1991 and 2018 also declined globally. The median age at first sexual intercourse among women aged 25–49 increased from 16 to 17 years showing that there was about a one-year increase in the age of sexual debut for young women over nearly three decades. Similarly, the median age at first marriage among women aged 25–49 years fell by 3.5%. This was associated with a concurrent decrease in the percentage of young men and women aged 15–24 between 1998 and 2018 who had sex before age 18.

Physical or sexual violence against women committed by husbands/partners reduced by approximately 7% between 2004 and 2018 thus reducing the exposure of women to HIV infection.

The proportion of women who are literate increased by 5.4% between 2004 and 2018 with a potential to increase knowledge on HIV prevention over this period.

The proportion of women who are in polygamous unions decreased by 16% between 1991 and 2018.

Between 2004 and 2018, the proportion of men and women receiving an HIV test and receiving test results in the last 12 months increased by 27.7% and 35.6% respectively; meanwhile the percentage of pregnant women aged 15–49 who received counselling, testing, and results on HIV during prenatal care increased by 17% reflecting an increasing number of PLWHIV who know their status and are then able to access treatment.

Between 1998 and 2018, the percentage of young men and women aged 15–24 years who reported having had sexual intercourse with more than one partner in the past 12 months reduced by 15.3% and 6.6% respectively; while in the same period, the percentage of young men and women aged 15–24 years who had more than one partner in the past 12 months and reported having used a condom during the last sex act increased rapidly between 1998 and 2014 by 24.6% and 26.3% respectively but did not change significantly thereafter.

**Table 1:** Trends in behavioural and social determinants of HIV in Cameroon between 1991 and 2018

HIV Indicators	CDHS 1991	CDHS 1998	CDHS 2004	CDHS 2011	CDHS 2018	Coeff (β)*	95% Confidence Interval	P-value
The population of Cameroon (million)	11.7	14.3	17.4	19.4	25.8	0.50	0.3–0.7	0.004
Median age at first sexual intercourse among women aged 25–49 (years)	16.0	15.8	16.4	17.0	17.0	0.05	0.01–0.09	0.03
Percentage of young women aged 15–24 who have had sex before age 18 (%)		79.0	71.0	62.0	62.0	-0.9	-1.9–0.1	0.06
Percentage of young men aged 15–24 who have had sex before age 18 (%)		52.0	45.0	44.0	35.0	-0.8	-1.4 - -0.1	0.04
Physical or sexual violence committed by husband/partner (%)			43.0	51.1	36.1	-0.5	-12.6–11.6	0.70
Literate women (%)			64.8	69.2	70.1	0.4	-1.4–2.2	0.23
Women who are in polygamous unions (%)	38.0	33.0	30.0	25.0	22.0	-0.6	-0.7 - -0.5	0.0002
Median age at first marriage among women aged 25–49 (years)	16.5	17.4	17.6	18.5	20	0.12	0.07–0.17	0.001
Women receiving an HIV test and receiving test results in the last 12 months (%)			4.8	22.0	40.4	2.5	1.9–3.2	0.01
Men receiving an HIV test and receiving test results in the last 12 months (%)			6.7	20.0	34.4	2.0	1.4–2.5	0.01
Percentage of pregnant women aged 15–49 who received counselling, testing, and results on HIV during prenatal care (%)				38.0	55.0	2.4	-	-
Percentage of young women aged 15–24 years who reported having had sexual intercourse with more than one partner in the past 12 months (%)		10.6	6.6	6.0	4.0	-0.3	-0.62–0.02	0.06
Percentage of young men aged 15–24 years who reported having had sexual intercourse with more than one partner in the past 12 months (%)		38.3	28.4	29.0	23.0	-0.7	-1.6–0.3	0.09
Percentage of young women aged 15–24 years who had more than one partner in the past 12 months and reported having used a condom during the last sex act (%)		17	41.6	37.0	37.0	0.8	-2.2–3.8	0.38
Percentage of young men aged 15–24 years who had more than one partner in the past 12 months and reported having used a condom during the last sex act (%)		30	56.3	43.0	40.0	0.2	-3.6–3.9	0.84

*β = Regression coefficients obtained from fitting a simple linear regression model

### Access to universal antiretroviral therapy and the number of people living with HIV

The number of people accessing antiretroviral therapy in Cameroon increased steadily from 17,156 to 2005 to 168,249 in 2015 and then exponentially to 337,342 in 2019 corresponding to a steady rise in ART coverage from 20% to 2010 to 40% in 2016 and then to a sharp increase to approximately 80% in 2021. This sharp increase from 2016 corresponded with the introduction of the test and treat-all strategy whereby every person living with HIV should receive ART as soon as possible irrespective of their immune status based on their CD4 counts. The increasing number of people receiving ART matched with an increasing number of people living with HIV, the fall in incidence, mortality, and prevalence (Fig. 3).

## Discussion

### Observed changes in HIV prevalence

In the last three septennial DHS surveys that included HIV testing, we have observed a consistent decline in the prevalence of HIV in Cameroon at a rate of about 1.35% every septennium. As a rule, at least three data points showing a steady trend in prevalence were needed to conclude there is a declining trend. The 95% confidence intervals around the point prevalence did not overlap indicating that the decline was statistically significant. The survey sample sizes were not less than three thousand households, the point estimates were reliable given the tight 95% confidence limits and the HIV prevalence rates were nationally representative. So, the observed fall in prevalence is valid in a statistical sense. These data owe their validity to the thorough and robust methodology intrinsic to the DHS Program portrayed by high standards in study planning and implementation; from sample design, developing and field-testing survey instruments, training of survey personnel, and careful supervision of data collection and processing. Estimates of HIV prevalence from national population-based surveys are considered the gold standard, especially in countries with relatively high prevalence (at least 2–3%) [14]. Results from the DHS are consistent with those obtained from two sentinel surveillance surveys (SSS) conducted among pregnant women in Cameroon. Though less reliable than population-based surveys, SSS are a good reflection of the latter in monitoring HIV dynamics. One of these SSS was conducted in 2016 among pregnant women from 60 sites in the ten regions of Cameroon, HIV prevalence among pregnant women declined significantly from 2009 (7.6%), 2012 (7.8%) to 2016 (5.7%), p < 0.0001 [15, 16]. Another SSS conducted in 2008 using data from seven sites in two regions of the country indicated that HIV prevalence decreased significantly in women younger than 20 years from 13% to 2000 to 5% in 2006 [17]. Similarly, HIV epidemiological updates, based on Estimations and Projections Package (EPP) and Spectrum that fit prevalence curves to surveillance and survey data, also revealed a decreasing epidemic in the general population from 5.3% to 2009 to 3.8% in 2016 [18, 19]. Reduction in HIV prevalence is occurring in other countries too [10, 20, 21]. A multi-country analysis of data from thirty countries most affected by the AIDS epidemic revealed that, out of the twenty-one countries that have data to assess national trends in HIV prevalence among 15–24-year-olds in recent years, the majority showed declines in HIV prevalence, and in ten countries statistically significant declines of more than 25% had occurred [10]. Therefore, there is sufficient evidence that the decline in HIV prevalence in Cameroon is genuine.

However, the main limitations of population-based surveys are the potential for bias introduced by sampling error, laboratory error, nonresponse, and the exclusion from the sampling frame of population groups at substantial risk of HIV infection [14, 22]. The validity of HIV prevalence results may change over time due to improvements in HIV tests per se and the implementation of laboratory quality assurance systems [11]. Starting around 2015 The DHS Program changed the HIV testing algorithm to add a confirmatory test for all EIA-positive specimens. A confirmatory test has high specificity to exclude some HIV-positive individuals and thus reducing the number of previously HIV-positive individuals detected by a single screening test with high sensitivity. This change has most likely affected the trend in HIV prevalence in Cameroon by overestimating the seroprevalence points derived from the surveys in 2004 and 2011.

HIV prevalence surveys may suffer from bias introduced by non-response, as non-responders may have distinct levels of HIV infection compared to those that participated and accepted an HIV test in the survey [22]. While people who refuse to take an HIV test in a survey may have higher levels of HIV infection than those who accept to take the test, there is also evidence to suggest that absence from a household is associated with increased HIV prevalence. People who travel and families affected by labour migration have higher HIV prevalence rates than others. Short-term mobility (traders, businesspeople, and people in search of work) may also be important, and people making frequent short trips may not be available during the time the survey team visits the household [23]. In Cameroon, the response rates for HIV testing for women were 92% and 90% for men in 2004 while in 2018 it achieved 98% for both men and women. The predicted prevalence of HIV among non-responding women and men was respectively 16% and 32% higher than among those tested for HIV in the 2004 survey. After adjusting the observed national estimates of HIV prevalence from tested men and women by accounting for the predicted rates among the non-responders, there was only a minor difference to the observed estimates probably because of the low non-response rates. Even in countries where the predicted prevalence among the non-responders was substantially higher than the observed prevalence, the adjusted prevalence for all eligible respondents was about the same as the observed prevalence based only on the tested respondents. Bias due to absence also had little influence on the estimate of overall prevalence because of the small proportion of absentees [14, 22].

Moreover, adjustments for nonresponse did not account for any bias due to exclusion of population members not living in households, such as those living on the street or in institutions (e.g., prisons, boarding schools, military barracks, refugee camps and brothels). The survey-based estimates of HIV prevalence are likely to be underestimated to the extent that the prevalence of HIV in these “non-household” populations is higher than that in household populations but given that the proportion of non-household populations in the total population tends to be small, any effect of excluding these populations on the national estimates obtained from a household-based sample is likely to be small, except possibly in some regions [14, 23]. Therefore, these shortcomings associated with the HIV population-based surveys could at most affect the rate of decline (slope of trendline) but are not sufficient to disregard the observed overall downward trend between 2004 and 2018. The declining trend is thus valid from statistical and epidemiological standpoints.

### Reasons for the decline in HIV prevalence

To understand the factors underlying the decrease in HIV prevalence, it is helpful to simultaneously triangulate changes in the determinants of prevalence: incidence, behaviours, and mortality. In the endemic steady state, the prevalence of infection is simply the product of incidence and the average duration of the infection. However, in an epidemic setting, the prevalence-incidence relationship depends on the age of the epidemic.

But first, let us turn our attention to the natural history of the HIV epidemic. HIV prevalence in a given population does not increase indefinitely but saturates at some point in time. After the initial spread of HIV, the incidence may decline, resulting in lower prevalence. Because the timescale for epidemic saturation is likely to be faster than the timescale for HIV-associated mortality to increase, declines in incidence likely precede declines in prevalence. It depends on the basic reproduction number (R_0_), and the number of new infections caused by infected individuals in a fully susceptible population. The reproduction number (Rt) at time t changes as the epidemic progresses. First, the incidence and prevalence in high-risk groups may increase exponentially. As the epidemic spreads, the rate of contact with already infected people increases. This reduces the reproducibility of infection and slows the increase in incidence. Ultimately, the incidence will decline while the prevalence will continue to increase. Prevalence decreases or evens out only when HIV mortality among infected persons increases [7]. If the mortality rate is higher than the incidence rate, the prevalence will decrease until the two balance out and the prevalence remains constant. This pattern cannot be explained by three data points from our population-based surveys, but it is beautifully explained by data based on the Estimations and Projections Package (EPP) and Spectrum, which fit prevalence curves to surveillance and survey data. According to this mathematical modelling, Cameroon’s HIV prevalence increased rapidly during the early-to-mid 1990s, levelled off in the early 2000s and then declined.

Data from Sentinel Site Surveillance (SSS) also show that Cameroon went through a steady increase in HIV prevalence since the early 1990s. The median HIV infection among antenatal women in Cameroon’s two main cities (Yaoundé and Douala) rose from 1% to 1990, 3% in 1992 to 5% in 1996 and then up to 9% in 2000 before falling to 7.6% in 2009 and then further down to 5.7% in 2016 [15, 16, 24–27]. From this, it can be concluded that her last three 7-yearly CDHSs were conducted after the country went through periods of acceleration and stagnation of the epidemic. The first two CDHSs, conducted in 1991 and 1998, did not collect data on HIV prevalence. Similarly, estimates of HIV incidence peaked around 1998 (later than many East Southern African countries) and then declined gradually thereafter, mainly as part of the natural course of the epidemic, primarily due to the saturation of infection in high-risk populations [7]. Mortality also peaked after incidence peaked around 2004 and then began to decline again, albeit at a slower rate than incidence (incidence/mortality ratio of 0.84). Thus, the decline in HIV prevalence is due firstly to the approximately 50% decline in incidence between 2000 and 2012, and secondly to the decline in incidence and associated relatively high mortality from 2012 to the present.

The decline in HIV prevalence was not only due to natural processes but also due to positive changes in sexual behaviour observed at the same time: a 1-year delay in sexual debut, a 7% fall in sexual violence, a 16% decrease in polygamous partnerships, 15.3% decrease in multiple sex partners and 26.3% increase in condom use. These positive changes contribute to the cumulative risk of contracting and becoming infected with HIV, leading to lower HIV incidence. These changes arise from the ambitious response to HIV, political commitment at the highest level, the know-how and involvement of government and civil society actors, with the constant support of technical and financial partners. An unprecedented effort has been made in the use of new communication technologies to raise awareness and promote services (prevention via the internet and social networks), the adaptation of services to the specific needs of adolescents and young people from KPs, FSW and MSM according to norms and standards, Reinforcing the availability of services through the extension of sexual health units within NGOs and health facilities for better access of KPs to services that meet their specific needs, the extension of pre-exposure prophylaxis (PrEP) for HIV prevention among MSM and FSW, strengthening the skills of health workers on combined prevention around hotspots, the diversified testing approach (medicalised, community-based and self-testing) to extend test coverage and reach targets beyond 95-95-95 for KPs, and the strengthening of coordination and networking between the actors involved in the provision of combination prevention services in the intervention sites [28]. The trend in the annual spending on HIV control in Cameroon nearly doubled between 2007 and 2009 (from 18.05 to 34.64 billion XAF), remained relatively stable thereafter because of the global economic hardship until 2015, and then almost tripled in 2016, to attain a peak in 2017 at 65.62 billion XAF [28].

### Impact of “test and treat All” on HIV prevalence

Antiretroviral therapy (ART) has transformed HIV/AIDS from a fatal disease to a manageable chronic but incurable disease [9, 29]. Increased access to ART also needs to be considered in the context of changes in HIV incidence and risky behaviour. The effect of ART use on the epidemic is reduced mortality and incidence. However, the availability of treatment in high-risk groups and the potential increase in risky behaviour due to complacency have been shown to negatively affect the magnitude by which these parameters are reduced Therefore, potential trade-offs between therapeutic benefits and adverse effects should be carefully monitored. People living with HIV have life expectancies similar to those of HIV-negative people if they are diagnosed early, have good access to medical care, and can adhere to HIV treatment [30]. A “test and treat” strategy means starting HIV treatment as soon as possible after HIV infection before CD4 cell counts drop to low levels. The sooner a diagnosis is made, and HIV treatment is started, the better the long-term prospects. People diagnosed in recent years (the era of “universal testing and treatment”) are more likely to live longer than those diagnosed recently. Living longer (reduced mortality) should lead to increased prevalence. This is because the incidence-prevalence ratio (IPR) remained above a threshold of 0.03 during the population-based survey, and the incidence of new infections was not zero though it was decreasing. IPR is defined as the number of new infections occurring each year in a population divided by the number of people living with HIV in the same population [31]. The epidemic will subside once the IPR drops below a certain threshold. If the IPR remains below this threshold for an extended period, the epidemic will eventually resolve. The IPR is a measure of progress in responding to the epidemic rather than having achieved the goal of ending the AIDS epidemic. Observed declines in prevalence, incidence, mortality and behavioural trends have become more pronounced since 2000, corresponding to an era when national and global commitments to combat the epidemic were gaining momentum.

So, if the prevalence of HIV (P) continues to decline even though the number of people living with HIV is increasing (Fig. 3), then what has offset the basic equation of *prevalence = (new cases + existing cases) / Population*? Referring then to the equation, new cases are declining, as indicated by the decline in incidence, and existing cases are declining from death at about the same rate, as indicated by the incidence-to-mortality ratio. Thus, the net increase in all cases (the numerator) remained relatively constant after the first DHS in 2004. Conversely, in the denominator, Cameroon’s population is growing at an exponential rate of more than 2% per year (approximately 500,000 people per year). This is due to the high fertility rate peculiar to developing countries. A growing population, mostly composed of young people, will ultimately dilute the growing number of people living with HIV, who are mostly adults, thereby contributing to the decline in HIV prevalence. Literature on the impact of HIV/AIDS on demographics is rich, but there is virtually no documentation on the impact of population growth on HIV prevalence. However, it is intuitive to point out that, despite an increase in the number of people living with HIV, the observed decline in HIV prevalence is largely due to the growing population of HIV-uninfected newborns and adolescents.

## Conclusion

The observed decline in HIV prevalence is statistically valid and reflects the observed decline in risky sexual behaviour that need to be sustained by the National HIV programme. Although universal access to ART has increased the number of persons surviving and ageing with HIV, it has not led to increased HIV prevalence in a setting with exponential population growth driven by young people with low HIV prevalence.

## Data Availability

Data can be obtained from the DHS Program upon request from archive@dhsprogram.com. and free from the UNAIDS website: https://www.unaids.org/en/resources/documents/2022/HIV_estimates_with_uncertainty_bounds_1990-present.

## Abbreviations

HIV: Human Immunodeficiency Virus
AIDS: Acquired Immune Deficiency Syndrome
ART: Antiretroviral Therapy
DHS: Demographic and Health Survey
UNAIDS: The Joint United Nations Programme on HIV/AIDS
CDHS: Cameroon Demographic and Health Survey
DBS: Dried Blood Sample
SPP: Spectrum Projection Package
EPP: Estimation and Projection Package
PLHIV: Persons living with HIV
95%CI: 95% Confidence Interval
SSS: Sentinel Surveillance Surveys
KP: Key Population
FSW: Female Sex Worker
MSM: Men who have Sex with Men
IPR: Incidence to Prevalence Ratio
CD4: Cluster of Designation 4
UTT: Universal Test and Treat
